# The Production of Recombinant Azurin from *Pseudomonas aeruginosa* and Its Ability to Induce Apoptosis in Various Breast Cancer Cell Lines

**DOI:** 10.3390/ijms26136188

**Published:** 2025-06-27

**Authors:** Tomasz Kowalczyk, Anna Merecz-Sadowska, Ewelina Synowiec, Tomasz Śliwiński, Janusz Piekarski, Janusz Szemraj, Mattia Mori, Patricia Rijo, Przemysław Sitarek

**Affiliations:** 1 Department of Molecular Biotechnology and Genetics, University of Lodz, 90-237 Lodz, Poland; 2 Department of Economic and Medical Informatics, University of Lodz, 90-214 Lodz, Poland; anna.merecz-sadowska@uni.lodz.pl; 3 Department of Molecular Genetics, University of Lodz, 90-236 Lodz, Poland; ewelina.synowiec@biol.uni.lodz.pl (E.S.); tomasz.sliwinski@biol.uni.lodz.pl (T.Ś.); 4 Department of Surgical Oncology, Medical University in Lodz, 93-513 Lodz, Poland; janusz.piekarski@umed.lodz.pl; 5 Department of Medical Biochemistry, Medical University of Lodz, 92-2156 Lodz, Poland; janusz.szemraj@umed.lodz.pl; 6 Department of Biotechnology, Chemistry and Pharmacy, University of Siena, 53100 Siena, Italy; mattia.mori@unisi.it; 7 CBIOS—Research Center for Biosciences & Health Technologies, Universidade Lusófona de Humanidades e Tecnologias, 1749-024 Lisbon, Portugal; patricia.rijo@ulusofona.pt; 8 iMed.ULisboa—Research Institute for Medicines, Faculdade de Farmácia da Universidade de Lisboa, Av. Prof. Gama Pinto, 1649-003 Lisbon, Portugal; 9 Department of Medical Biology, Medical University of Lodz, 90-151 Lodz, Poland; przemyslaw.sitarek@umed.lodz.pl

**Keywords:** azurin, anticancer, apoptosis, breast cancer, recombinant proteins

## Abstract

Azurin is a copper-containing redox protein naturally produced by *Pseudomonas aeruginosa*, which has shown promising activity against human cancer cells by inducing apoptosis. The present study describes the design of a recombinant vector, pT7-MAT-Tag-2-Azu, for azurin production in *E. coli* cells. The cytotoxic effects of purified azurin were tested on three breast cancer cell lines (MCF-7, MDA-MB-231, and HCC38) and a normal breast epithelial cell line (MCF10A) using the MTT assay. The results showed cytotoxicity against cancer cell lines with minimal effects on normal cells. Further analysis showed that azurin induced apoptosis through mitochondrial pathways, as evidenced by increased expression of apoptosis-related genes (*Bax*, *TP53*, *Apaf-1*, *caspase-3, -8, -9*) and their corresponding proteins, elevated levels of reactive oxygen species (ROS), and DNA damage, mitochondrial membrane potential (MMP), or brine shrimp lethality assay. Furthermore, in silico molecular docking, simulations predicted a stable, electrostatically driven interaction between azurin and the p53 protein, providing a structural basis for its mechanism of action. These findings suggest that recombinant azurin may serve as a potential therapeutic agent for breast cancer after further multifaceted research.

## 1. Introduction

Among the many achievements of modern molecular biotechnology, one extremely important branch involves the production of recombinant proteins using the overexpression of foreign genes in various host organisms. The precise and efficient strategies currently in use for manipulating genetic material are opening up new possibilities in many fields of human life. A wide range of precision tools currently in use, including various vectors and expression systems, has facilitated the production of a wide spectrum of proteins in large quantities that can be used in basic or applied research.

The introduction of the first recombinant protein with medical application—insulin—into widespread use in 1982 has since been followed by a wide range of proteins with various applications [1]. Currently available expression systems for the production of recombinant proteins differ in to their level of complexity, costs, efficiency, and the possibility of carrying out post-translational modifications. Each of these systems has its own specific features that determine its suitability for a specific application. The most commonly used expression platforms today are bacterial, yeast, mammalian, and plant cells, although bacterial systems are often chosen as they offer economic efficiency, well-characterized molecular mechanisms, ease of genome manipulation, growth speed, simplified product purification procedures, and easy scalability. It is worth emphasizing that each system can also be used on a large scale thanks to modern and strictly controlled technical solutions [2,3,4].

Among the huge number of proteins obtained by recombinant DNA, including blood coagulation factors, various monoclonal antibodies, erythropoietin, vaccine proteins, and industrial proteins [4,5,6], those showing a wide spectrum of desired properties are of particular interest, with the most noteworthy being those of bacterial origin. Many of them show highly desirable properties, such as bacteriocins, which are widely used in food preservation or as an important alternative to antibiotics [7,8]. One noteworthy example is azurin. This copper-containing redox protein is naturally produced by the pathogenic bacterium *Pseudomonas aeruginosa*. Azurin, about 14 kDa in size, plays a key role in the electron transport chain of some bacteria, acting as a redox protein. It is a type of cupredoxin involved in electron transfer between different molecules involved in cellular respiration. In addition, the stability of azurin and its ability to undergo redox reactions without losing structural integrity have made it a focus of interest beyond its biological role [9,10]. Research into its potential applications in, for example, biotechnology or medicine, is particularly warranted, not least because of its potential use in biosensors and bioelectronic devices [11]. Indeed, there is a considerable body of evidence that confirms its interesting and medically significant anticancer properties [12]. The World Health Organization’s International Agency for Research on Cancer (IARC) predicts that there will be more than 35 million new cases of cancer by 2050—an increase of 77% compared to 2022 statistics [13]. The reports emphasize that such a rapid increase necessitates the need for increased global efforts in the prevention, early diagnosis, and effective treatment of the disease. Of these, one of the greatest threats appears to be breast cancer. In 2020, this cancer was the most commonly diagnosed cancer worldwide (more than 2.26 million new cases) and is now the second most common type of cancer, after lung cancer. There is hence an urgent need to develop effective strategies for its treatment [14].

Studies have found azurin to demonstrate its anticancer activity by selectively penetrating into human cancer cells, where it exhibits both cytostatic and cytotoxic effects. Experiments involving truncation showed that the entry of azurin into these cancer cells is facilitated primarily by a 28 amino acid segment known as p28. Due to these properties of azurin, it is an interesting candidate for further studies of anticancer properties, especially as it shows preferential selectivity for cancer cell lines, exhibiting significantly reduced cytotoxic and apoptotic activity in normal cell lines [15].

The anticancer mechanisms of azurin are multifaceted, but one of the most studied pathways involves its direct interaction with the tumor suppressor protein p53. Studies have shown that azurin, particularly through its p28 domain (a 28-amino-acid segment), can preferentially enter cancer cells and form a stable complex with the DNA-binding domain of p53 [16,17]. This interaction is believed to stabilize p53 by preventing its ubiquitination and subsequent proteasomal degradation. The resulting increase in intracellular levels of functional p53 triggers the mitochondrial apoptotic pathway, characterized by an altered balance of pro-apoptotic (e.g., Bax) and anti-apoptotic (e.g., Bcl-2) proteins, ultimately leading to the activation of the caspase cascade (e.g., caspase-9 and caspase-3) and programmed cell death [18,19].

However, the clinical applicability of azurin as a p53-targeting agent is complicated by the high frequency of mutations in the *TP53* gene. These mutations are a critical driver in tumorigenesis, and cancer genome sequencing has shown that 42% of cases across 12 major tumor types carry a mutant *TP53* gene [20]. In breast cancer, *TP53* is the most frequently mutated gene, with mutations found in nearly 30% of all cases. Importantly, this frequency varies significantly across molecular subtypes. For example, *TP53* is mutated in nearly 80% of triple-negative breast cancers (TNBCs), while its incidence in HR-positive breast cancers is approximately 20% [21]. This heterogeneity underscores that the efficacy of azurin may be dependent on the p53 status of the tumor. Therefore, a thorough understanding of this interaction and the molecular diagnosis of p53 status are crucial prerequisites before considering azurin for clinical trials [22].

In the present study, a recombinant pT7-MAT-Tag-2-Azu vector was constructed to overproduce recombinant azurin in *E. coli* cells, which was then used to test breast cancer cell lines MCF-7, MDA-MB-231, and HCC38 for their ability to induce apoptosis through mitochondrial pathways and to affect the expression of proapoptotic and anti-apoptotic genes and protein levels. The effects of azurin on ROS levels and DNA damage in cancer cells were also tested.

## 2. Results

### 2.1. Obtaining the Recombinant Expression Vector

**Figure 1:**
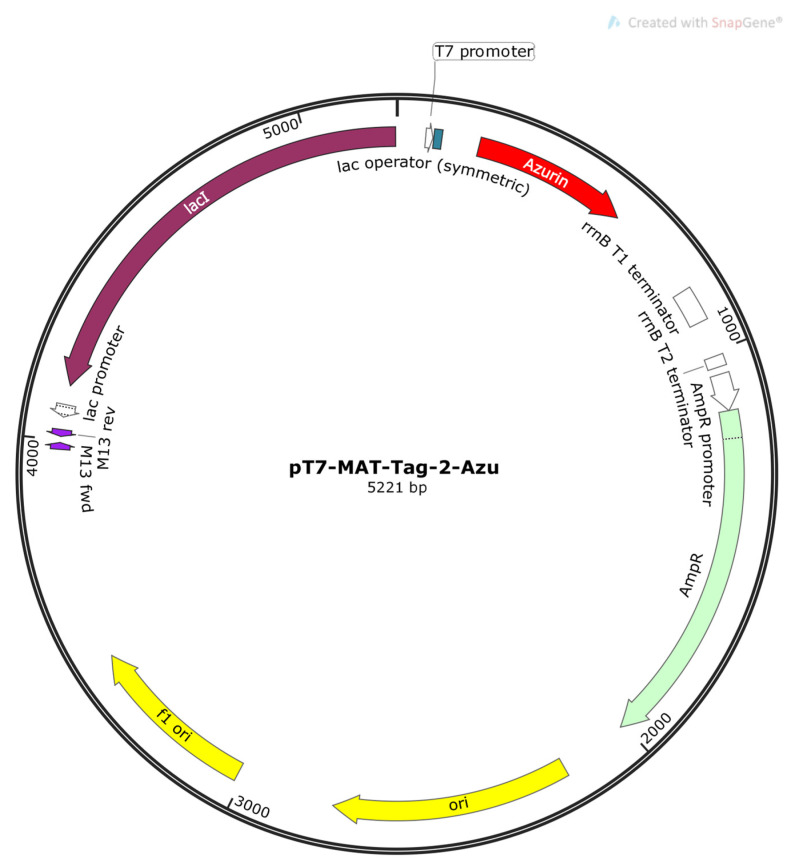
Recombinant vector pT7-MAT-Tag-2-Azu (SnapGene^®^ software, www.snapgene.com, ver. 4.2.11).

The azurin sequence was successfully inserted into the vector pT7-MAT-Tag-2-Azu, whose structure is given in Figure 1.

### 2.2. HADDOCK Docking Predicts a Stable Azurin-p53 Complex Driven by Electrostatic Interactions

To provide a structural model for the azurin-p53 interaction, in silico protein–protein docking was performed. The HADDOCK simulation yielded a highly converged and well-defined structural cluster, which was ranked as the best solution. This top-ranked cluster was significantly populated, containing 99 of the 200 water-refined models, and its structures were highly consistent, with a low root-mean-square deviation (RMSD) of 0.9 ± 0.5 Å from the overall lowest-energy model.

The cluster exhibited a favorable HADDOCK score of −79.0 ± 3.5. The statistical significance of this result was confirmed by a Z-score of −1.5, indicating that the predicted binding mode is reliable and not a result of random chance. The analysis of the energy contributions showed that the interaction is predominantly driven by strong electrostatic forces (E_elec = −249.5 ± 23.8 kcal/mol), which was complemented by significant van der Waals interactions (E_vdw = −45.4 ± 2.7 kcal/mol), indicating a high degree of shape complementarity. The formation of the complex led to a large buried surface area (BSA) of 1444.1 ± 77.4 Å^2^, which is characteristic of a stable and specific protein–protein interface. While the desolvation energy was slightly unfavorable (6.6 ± 1.3 kcal/mol), as is typical for such interactions, a moderate restraints violation energy (96.5 ± 7.3 kcal/mol) suggests that minor conformational adjustments occurred to achieve the optimal energetic fit.

**Figure 2:**
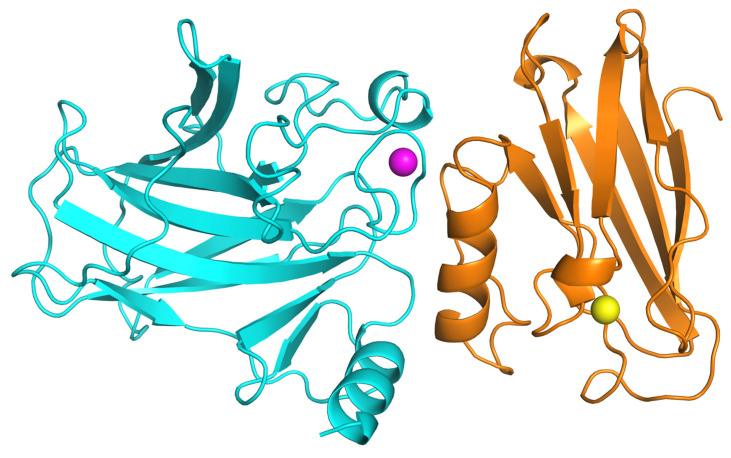
Structural model of the Azurin-p53 complex predicted by HADDOCK. The top-scoring model from the best-ranked cluster is shown in a cartoon representation. Azurin (orange) is docked into a surface groove of the p53 DNA-binding domain (cyan). The catalytic copper ion (Cu^2^⁺) in azurin is shown as a yellow sphere, and the structural zinc ion (Zn^2^⁺) in p53 is shown as a magenta sphere. The image was generated using PyMOL (version 3.1).

The lowest-energy structural model from this cluster is presented in Figure 2. Visual analysis of the binding pose reveals that the p28 domain of azurin docks into a surface-exposed groove on the p53 DNA-binding domain, consistent with the active residues defined for the simulation. Overall, these in silico results provide a robust structural model that strongly supports a direct and energetically favorable interaction between azurin and p53, thereby offering a molecular basis for its pro-apoptotic activity.

### 2.3. Analysis of the Correctness of the Recombinant Vector Construction

The isolated plasmids after genetic transformation of competent *E. coli* cells were analyzed for construct validity. PCR analysis of the isolated plasmid DNA revealed the presence of an amplification product of the gene encoding azurin. An amplification product of 387 bp was observed, which confirms the presence of the sequence encoding azurin in the recombinant plasmid. In addition, hydrolysis of the plasmid by HindIII and KpnI also revealed the presence of a fragment of similar size.

**Figure 3:**
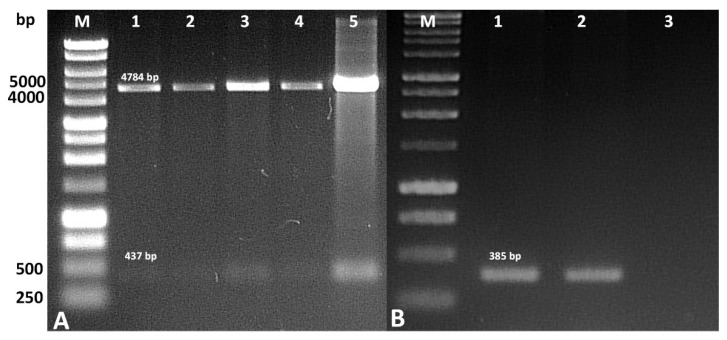
Result of analysis of the pT7-MAT-Tag-2-Azu recombinant vector construct. (**A**) Hydrolysis by restriction enzymes, M-DNA size marker, 1–5—results of hydrolysis of independent recombinants; (**B**) PCR analysis, M-DNA size marker, 1–2—PCR on recombinant vectors, 3—negative control without vector.

Hydrolysis of the recombinant vector during the vector construction stage (HindIII and KpnI) revealed the presence of a fragment 437 bp in length. Similarly, an amplicon of similar size was revealed by the PCR reaction, confirming the presence of a gene encoding azurin in the recombinant plasmid (Figure 3).

### 2.4. Expression of Recombinant Azurin in E. coli DE3 Rosetta

**Figure 4:**
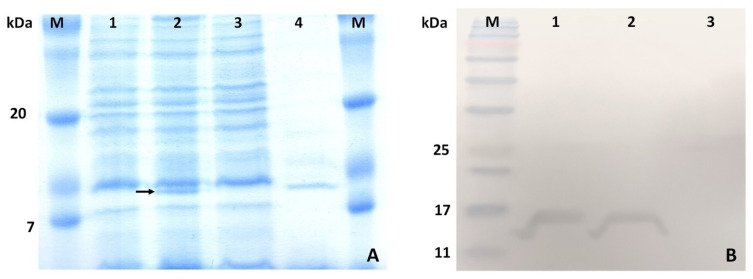
(**A**) SDS-PAGE of protein isolated from transformed *E. coli*, M-Protein ladder, 1—proteins isolated from non-IPTG-induced bacteria, 2—proteins isolated from IPTG-induced bacteria, 3—flow through (IMAC purification), 4—purified azurin; (**B**) Western blot analysis of affinity chromatography purified recombinant azurin, M—prestained protein marker, 1–2—purified recombinant azurin (independent cell lysates), 3—negative control (isolated proteins from untransformed *E. coli* cells).

Expression of recombinant azurin in *E. coli* DE3 Rosetta was induced with IPTG. The results were monitored by SDS-PAGE analysis. After optimization the greatest productivity of *E. coli* cells was obtained with 0.5 mM IPTG and culture at 28 °C for 12 h. In induced transgenic bacterial cells, a band corresponding to the expected molecular weight of azurin (~15 kDa) was observed, which was not present in untransformed cells. The results obtained indicate successful production of azurin in *E. coli* cells (Figure 4A).

### 2.5. Purification and Confirmation of Recombinant Azurin

**Figure 5:**
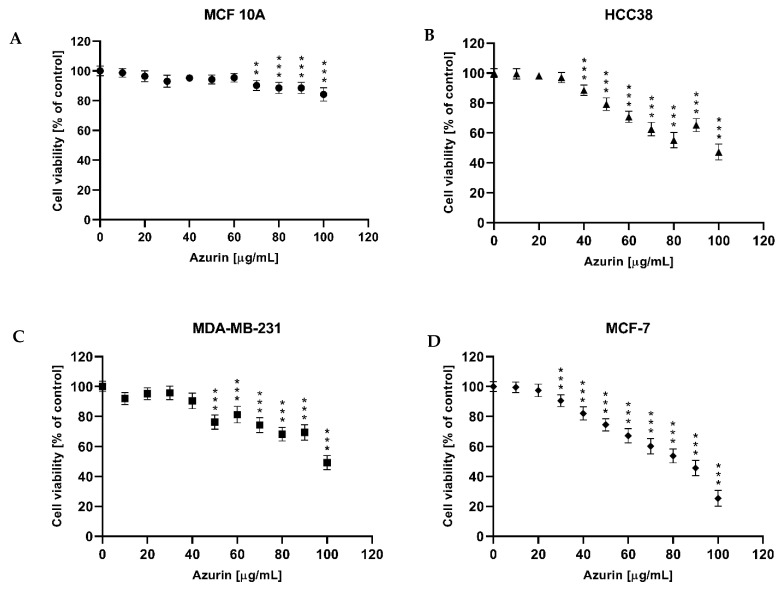
Viability of the normal MCF10A cell line (**A**), and the breast cancer cell lines HCC38 (**B**), MDA-MB-231 (**C**), and MCF-7 (**D**) after treatment with recombinant azurin. Cells were treated with various concentrations of recombinant protein. The cytotoxic effect was tested after 24 h of exposure. Data shown are the mean values of three independent experiments. ** *p* < 0.01, *** *p* < 0.001.

Recombinant azurin was purified by immobilized metal ion affinity chromatography using a C-terminal 6xHis tag. As a result, a highly purified protein was obtained, as evidenced by the presence of a single band on SDS-PAGE. The recombinant azurin was confirmed using Western blot: a single band at the expected molecular mass with a 6xHis tag was observed (Figure 5B).

### 2.6. MTT Cell Viability Assay

The MTT assay was performed to test the cytotoxic effect on three breast cancer cell lines MCF-7 (human breast; mammary gland; adenocarcinoma), MDA-MB-231 (human breast cancer cell line), and HCC38 (human breast cancer cells) and the normal cell line MCF10A (Figure 4). It was found that after 24 h exposure in the tested concentration range (0–100 µg/mL), the recombinant azurin demonstrated different IC_50_ values against HCC38 breast cancer cell line (100.77 µg/mL; Figure 5B), MDA-MB-231 (121.69 µg/mL; Figure 5C), and MCF-7 (81.01 µg/mL; Figure 5D). In contrast, no cytotoxic effect was observed in the tested concentration range for the normal MCF10A line (Figure 5A).

### 2.7. Gene Analysis Using RT-PCR and Protein Level Determination

**Figure 6:**
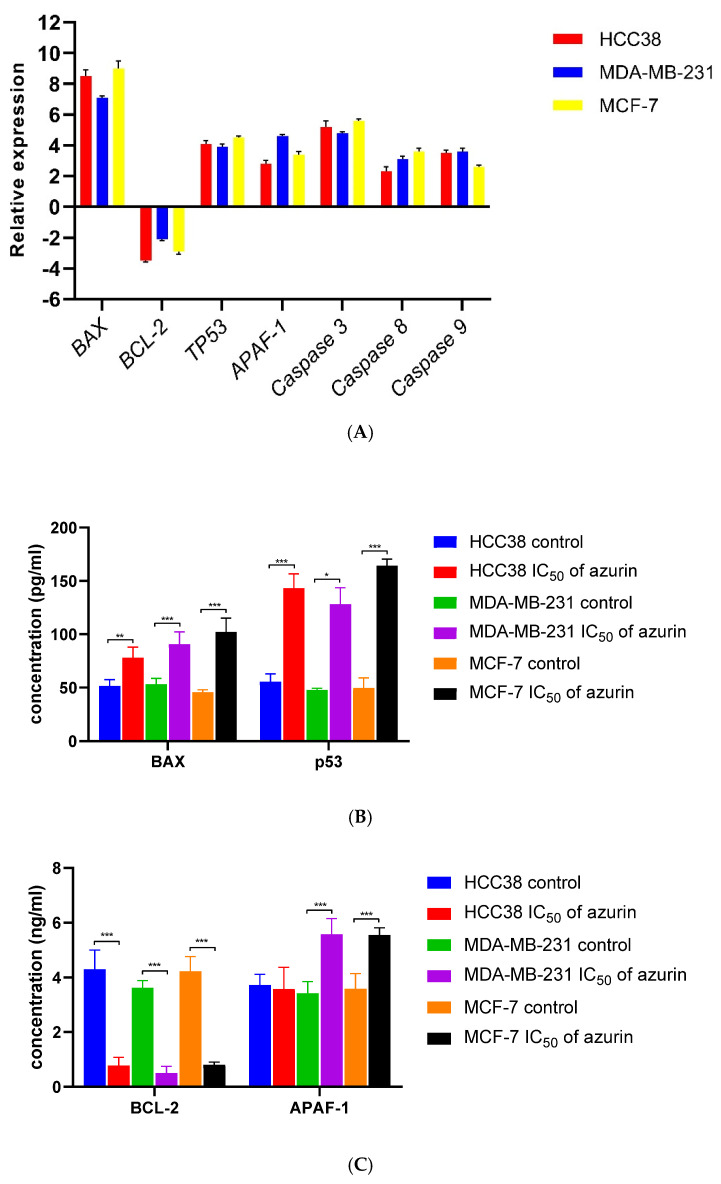
Effect of recombinant azurin on the expression of *Bax*, *Bcl*-2, *TP53*, *Apaf*-1, caspase-3, caspase-8, and caspase-9 in MCF-7, MDA-MB-231, and HCC38 cancer cells. Expression at the mRNA (**A**) and protein levels (**B**,**C**). Results are mean ± SD from three independent experiments. Protein levels of BAX, BCL-2, p53, and APAF-1 in HCC38, MDA-MB-231, and MCF-7 cancer cell lines determined by ELISA assay after treatment with recombinant azurin protein (IC_50_ for 24 h). Results are mean ± SD from four independent experiments. * *p* < 0.05, ** *p* < 0.001, *** *p* < 0.001.

A significant 7.20-to-8.30-fold increase in the expression of the *Bax* gene was observed in all treated cell lines (MCF-7, MDA-MB-231, and HCC38). In contrast, moderate increases of 2.8-to-4.25-fold were observed for the *TP53*, *Apaf-1*, and caspase-3, -8, and -9 genes, with the different cell lines demonstrating similar levels of expression (Figure 6A). In contrast, all tested lines exhibited a 2.2–4.25-fold decrease in *Bcl-2* gene expression (Figure 6A). Analysis of the levels of apoptosis-related proteins showed the strongest effect on the increase in p53 and BAX levels under the influence of recombinant azurin and a decrease for BCL-2, and the weakest effect on APAF-1 in the tested breast cancer cells (Figure 6B,C).

### 2.8. Comet Assay

**Figure 7:**
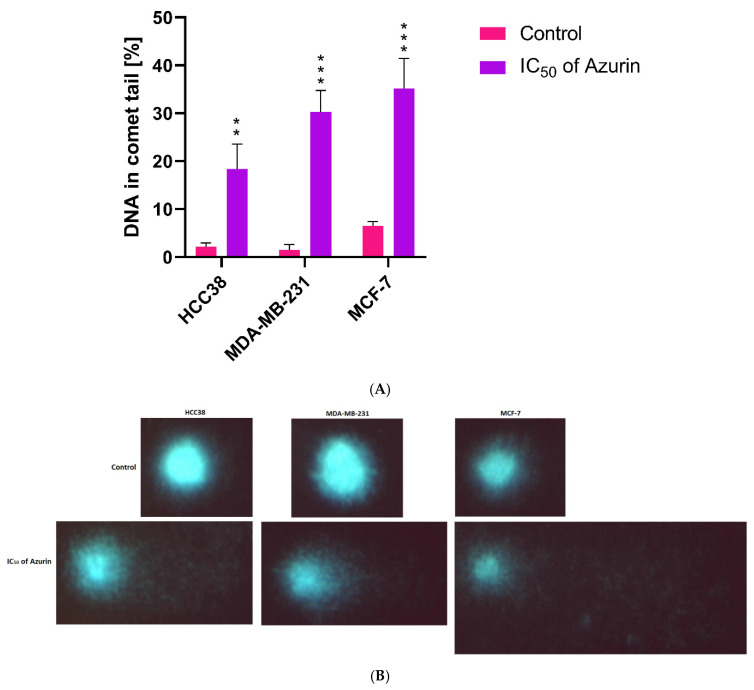
(**A**) Comet assay showing recombinant protein azurin-induced DNA breaks in MCF-7, MDA-MB-231, and HCC38 cells for 24 h. (**B**) In untreated MCF-7, MDA-MB-231, and HCC38 cells, a small amount of inherent endogenous DNA damage could be seen. Results are given as mean ± SD from three independent experiments. ** *p* < 0.01, *** *p* < 0.001.

The effect of recombinant azurin protein treatment on nuclear DNA damage was evaluated by quantitative single-cell gel electrophoresis assay (comet assay). An increase in DNA damage (indicated by increased comet tail formation) was observed in the treated MCF-7, MDA-MB-231, and HCC38 cells compared to untreated cells. All of the cell lines tested showed damage: approximately 19% in the HCC38 line, 29% in the MDA-MB-231 line, and 34% in MCF-7 (Figure 7A). Figure 7B shows representative images.

For the control HCC38 cells, the mean head DNA was 5767.244 ± 1221.045, and the mean tail DNA was 11.237 ± 24.547. In the case of control MDA-MB-231 cells, the mean head DNA was slightly higher at 5890.123 ± 1250.321, with a tail DNA of 15.456 ± 28.765. The MCF-7 control cells showed more baseline damage, with a mean head DNA of 5600.789 ± 1300.654 and a tail DNA of 25.678 ± 35.432. After treatment with IC_50_ concentrations of azurin, all cell lines exhibited increased DNA damage. For HCC38 cells, the mean head DNA decreased to 5577.886 ± 1150.621, while the tail DNA increased significantly to 491.547 ± 401.320. MDA-MB-231 cells showed more pronounced effects, with a mean head DNA of 5400.234 ± 1180.765 and a tail DNA of 580.678 ± 450.321. The MCF-7 cells were the most affected, with a mean head DNA of 5200.543 ± 1220.432 and a tail DNA of 650.789 ± 500.654.

The percentage of DNA in the tail, indicative of DNA damage, was approximately 19% in the HCC38 line, 29% in the MDA-MB-231 line, and 34% in MCF-7 (Figure 6A). Figure 6B shows representative images of the comet assay for each cell line in both control and treated conditions.

### 2.9. Reactive Oxygen Species Activation Assay

**Figure 8:**
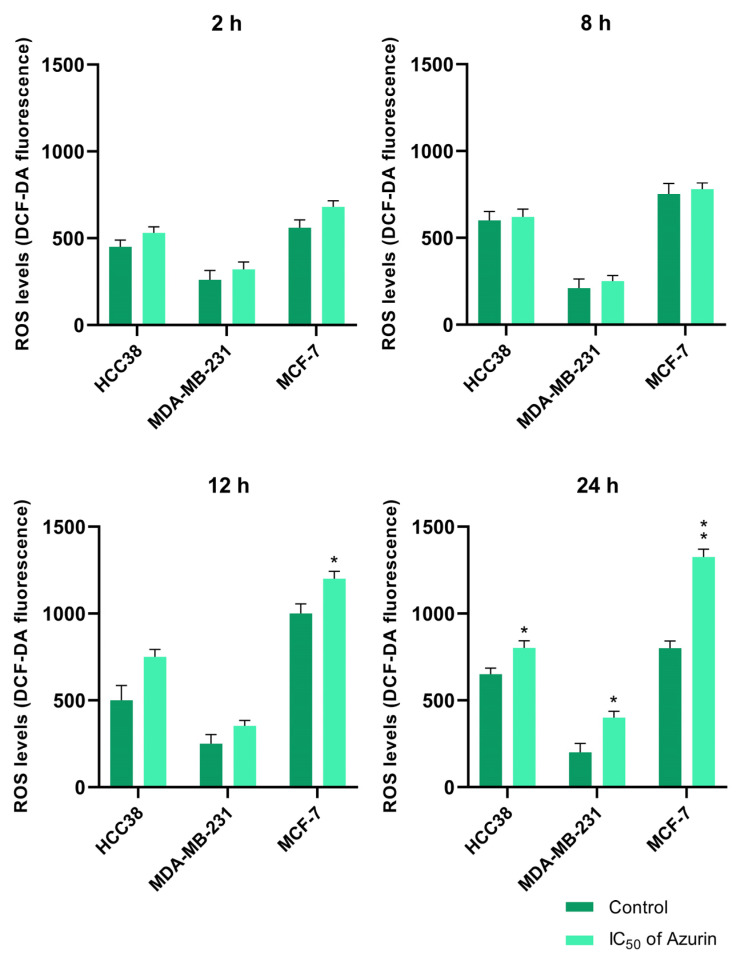
ROS levels of MCF-7, MDA-MB-231, and HCC38 cancer cells treated with the IC_50_ of recombinant azurin protein for 2, 8, 12, and 24 h along with controls were measured after staining with 2′,7′—Dichlorofluorescin diacetate (DCFH-DA). Data are given as the mean of three independent experiments. * *p* < 0.05, ** *p* < 0.01.

All cell lines (MCF-7, MDA-MB-231, and HCC38) treated with azurin at the IC_50_ for 2, 8, 12, and 24 h showed increased levels of ROS (Figure 8), with the greatest effect observed for the MCF-7 cell line. An increase in ROS levels is an indication of significant cell damage and programmed cell death. The mean intensity of the fluorescence was measured together with the control values.

### 2.10. Caspase 3/7 Activity Analysis

**Figure 9:**
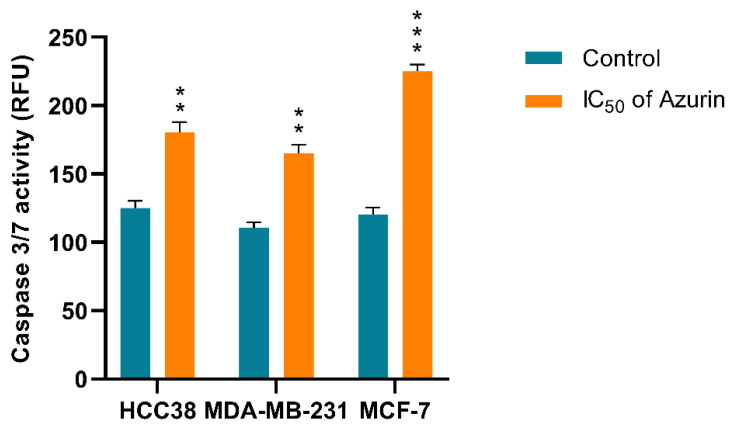
Caspase 3/7 activity (RFU) in MCF-7, MDA-MB-231, and HCC38 cancer cells treated with IC_50_ concentration of recombinant azurin protein for 24 h. The activities of caspase 3/7 in treated and untreated cells were then determined by luminescence assay. Data shown are the mean of three independent experiments. ** *p* < 0.01, *** *p* < 0.001.

As expected, all cell lines treated with azurin (the IC_50_ for 24 h) demonstrated increased caspase 3/7 activity, indicating the presence of apoptosis (Figure 9). The highest activity was observed in the MCF-7 line.

### 2.11. Azurin Induces Depolarization of the Mitochondrial Membrane

**Figure 10:**
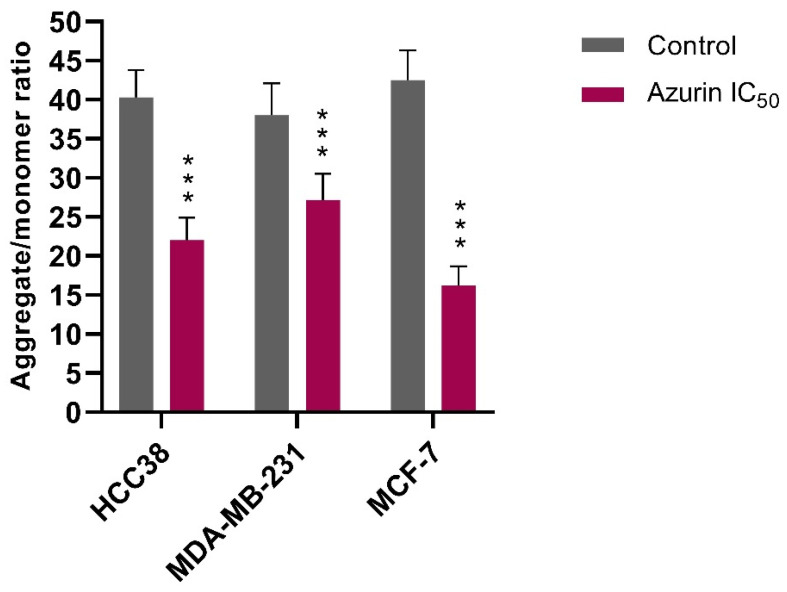
Effect of recombinant azurin on the mitochondrial membrane potential (ΔΨm) in breast cancer cells. MCF-7, MDA-MB-231, and HCC38 cells were treated with azurin at their respective IC_50_ concentrations for 24 h. The mitochondrial membrane potential was quantified using the JC-1 probe. Data are expressed as the mean ratio of red (J-aggregate) to green (JC-1 monomer) fluorescence ± SD from three independent experiments. Asterisks indicate a statistically significant decrease compared to the corresponding untreated control group (*** *p* < 0.001).

To investigate whether azurin-induced cytotoxicity is mediated by the mitochondrial apoptotic pathway, the change in mitochondrial membrane potential (ΔΨm) was assessed using the JC-1 fluorescent probe. Treatment with recombinant azurin at the respective IC_50_ concentrations for 24 h resulted in a significant loss of ΔΨm in all three breast cancer cell lines, as indicated by a decrease in the red/green fluorescence ratio (Figure 10).

The most pronounced effect was observed in the MCF-7 cell line, where the aggregate/monomer ratio significantly decreased from a control value of 42.5 ± 3.8 to 16.2 ± 2.5 following exposure to azurin. A significant reduction in the ratio was also documented for the HCC38 cells (from 40.3 ± 3.5 to 22.0 ± 2.9) and the MDA-MB-231 cells (from 38.1 ± 4.1 to 27.2 ± 3.3).

These findings demonstrate that recombinant azurin induces mitochondrial membrane depolarization in breast cancer cells. The magnitude of this effect varied between the cell lines, consistent with the previously observed differences in cytotoxicity.

### 2.12. Assessment of General Toxicity of Recombinant Azurin

**Figure 11:**
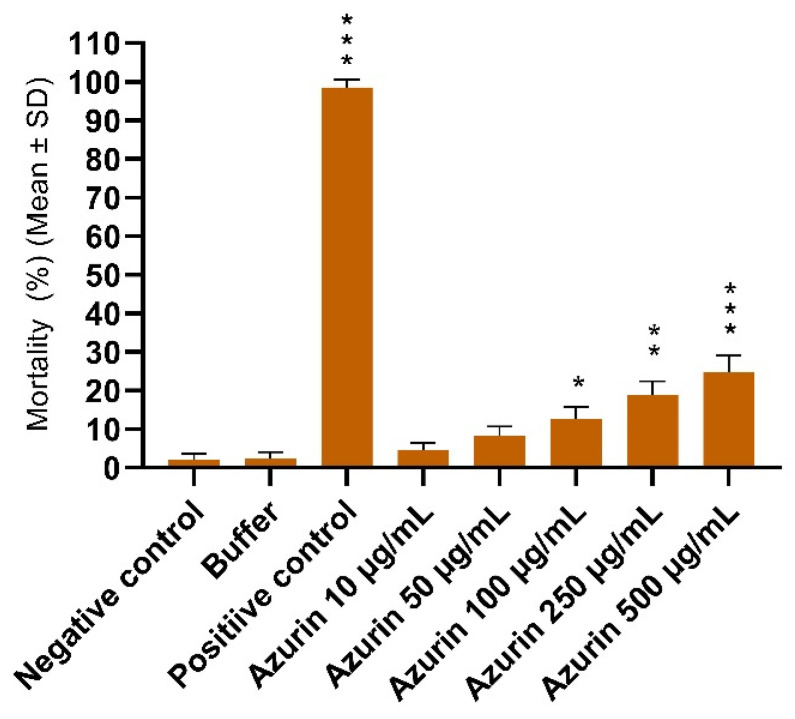
General toxicity of recombinant azurin evaluated by the brine shrimp (*Artemia salina*) lethality assay. Nauplii were exposed to a range of azurin concentrations for 24 h. The columns represent the mean percentage of mortality ± standard deviation (SD) from three independent experiments. A vehicle control (protein buffer) and a positive control (1 mg/mL potassium dichromate, K_2_Cr_2_O_7_) were included to validate the assay. Statistical significance was determined by comparing each azurin treatment group to the vehicle control group using a one-way ANOVA followed by Dunnett’s post hoc test. (* *p* < 0.05; ** *p* < 0.01; *** *p* < 0.001).

The general toxicity of recombinant azurin was evaluated using the brine shrimp lethality assay to assess its potential impact on a non-mammalian eukaryotic organism. The results indicated that azurin possesses very low toxicity. A slight, dose-dependent increase in mortality was observed with increasing concentrations of the protein (Figure 11).

At the highest tested concentration of 500 µg/mL, azurin induced a mortality rate of only 24.8 ± 4.2%. The negative and vehicle (buffer) controls exhibited minimal mortality (2.1 ± 1.5% and 2.3 ± 1.8%, respectively), confirming the viability of the nauplii and the non-toxic nature of the buffer. In contrast, the positive control (potassium dichromate) caused nearly complete mortality (98.5 ± 2.1%), validating the sensitivity of the assay.

Based on the established toxicity criteria for this assay, a substance is considered non-toxic or to have low toxicity if its LC_50_ (the concentration required to kill 50% of the population) is greater than 100 µg/mL. Since the mortality rate did not approach 50% even at the highest concentration, the LC_50_ value for recombinant azurin was determined to be >500 µg/mL. This finding suggests that recombinant azurin exhibits low general toxicity, which is consistent with its known selective mechanism of action against cancer cells.

## 3. Discussion

Cancer has long remained one of the greatest challenges of modern medicine. One potentially effective type of therapeutic strategy may involve the use of bacterial proteins. The literature data indicate that such proteins often have a unique ability to selectively eliminate cancer cells through various mechanisms of action [23,24,25]. Their activity is even more desirable because they often do not appear to have any negative effect on normal cells [16]. The bacterial proteins bovicin, colicins, nisin, and plantaricin may be active against very different types of cancer and are hence promising candidates in the fight against cancer while minimizing damage to healthy tissues [26,27,28,29].

One such antibacterial protein with anticancer properties that deserves special attention is azurin, which can be produced in various biological systems. The production of recombinant protein in *Escherichia coli* has become an extremely important element of modern medical biotechnology. This system offers the advantages of high efficiency and rapid cell growth, inexpensive production, ease of introducing genetic modifications, easy scalability, and well-developed and efficient purification methods. Azurin, produced by *Pseudomonas aeruginosa*, can be used to attack tumors, parasites, bacteria, or viruses [30]. Azurin is a 128 amino acid (14 kDa) copper-containing member of the cupredoxin family of redox proteins, which is secreted as a periplasmic protein. It has an elongated α-helix transduction domain (Leu50-Asp77) and four C-terminal loop regions, including CD, EF, FG, and GH loops, whose structure resembles the variable domains of immunoglobulin antibodies [15,31]. Numerous studies confirm that azurin is able to enter tumor cells and inhibit their growth; this leads to cell shrinkage and death through multiple mechanisms, including (i) binding to the DNA-binding domain (DBD) of the tumor suppressor protein p53, (ii) antiproliferative activity, and (iii) proapoptotic activity [17,32,33,34]. In addition, the protein is not toxic to healthy cells. Another important feature of azurin is its ability to act as a cargo protein, allowing many therapeutic agents to bind to its molecule [15,35,36].

Despite these promising anticancer properties, a critical consideration for the clinical application of azurin is its origin from *Pseudomonas aeruginosa*, a notorious opportunistic pathogen responsible for severe and often difficult-to-treat infections, particularly in immunocompromised individuals [37,38]. Consequently, legitimate concerns arise as to whether the exogenous administration of this recombinant protein could inadvertently support *P. aeruginosa* survival or virulence in the host, for instance, by supplementing its bacterial electron transport chain. It is crucial to emphasize, however, that therapeutic investigations, including the present study, utilize a highly purified, recombinant form of azurin, devoid of other bacterial virulence factors. Furthermore, the anticancer mechanism of azurin, attributed to its preferential entry into cancer cells (often mediated by the p28 domain) and its interaction with key eukaryotic regulatory proteins such as p53 [22,26], is distinct from its physiological role in bacterial metabolism. In evaluating the risk–benefit trade-off, the observed selective cytotoxicity against cancer cells, with minimal impact on normal cells (as also corroborated by our findings for the MCF10A line), appears highly advantageous. Furthermore, our study provides additional preliminary evidence supporting this favorable safety profile. The brine shrimp lethality assay, a widely used model for assessing general toxicity, revealed that the recombinant azurin possesses very low acute toxicity, with a calculated LC_50_ value greater than 500 µg/mL. This results in a whole-organism model that, while simple, reinforces the argument that the purified protein is unlikely to cause systemic toxicity, further distinguishing its therapeutic action from the pathogenicity of its source organism [39,40].

The aim of our work was to construct an appropriate plasmid vector for the production of recombinant azurin in transgenic *E. coli* cells and then to isolate, purify, and test its anticancer properties in relation to selected cancer cell lines of various breast cancer types in vitro. In this work, the plasmid expression vector pT7-MAT-Tag-2 was modified by inserting the gene encoding azurin previously excised from the pEX-K168-azu plasmid using restriction enzymes. The recombinant vector was introduced into *E. coli* BL21(DE3) Rosetta T1R cells to produce the recombinant protein. This is the first report of such a strategy to produce recombinant azurin in a Rosetta *E. coli* strain system, despite the previous use of *E. coli* to produce the protein.

In previous studies, Kha et al. [41] and Sereena et al. [34] report using *E. coli* with the T7 promoter to produce azurin based on the recombinant pET22b+ vector, while Aslam et al. achieved a satisfactory level of azurin production by cloning the gene into the pET20b vector with the T7 promoter [30]. A similar approach was presented by Mohammadi-Barzeligh et al., who obtained azurin expression in *Escherichia coli* BL21 based on the pET21 vector [42]. In contrast, Yildiz et al. used the Nisin Controlled Gene Expression (NICE) system to express azurin in *Lactococcus lactis* by using the recombining plasmid pNZ8149 [43]. The present study optimized the conditions for azurin production by testing the effect of key parameters such as IPTG concentration, bacterial culture temperature, and culture time. It was found that optimal recombinant azurin production was achieved using a lower concentration of IPTG and lowering the temperature during recombinant protein expression. Further diversifying the production strategies, Ma et al. recently demonstrated the efficient production and cytotoxic effect of recombinant azurin delivered via *Escherichia coli* Nissle 1917-derived minicells against colon cancer cells, highlighting another promising avenue for azurin-based therapies [44]. Such a strategy is frequently used to enhance protein overexpression in bacterial cells; it results in less protein aggregation and thus better protein folding, thus reducing abnormal products and consequently the deposition of protein products as inclusion bodies. Our results are consistent with those of Mohammadi-Barzeligh et al., who achieved optimal protein production by selecting the appropriate inducer concentration with extended culture time after IPTG induction [42]. In the present study, high yields of recombinant azurin were obtained and purified using His-Select Nickel Affinity Gel for affinity chromatography (IMAC). The isolated protein was then tested for anticancer properties. The use of IMAC resulted in highly pure protein, as evidenced by SDS-PAGE analysis. These findings are consistent with those of Kha et al., who successfully purified azurin using a Ni^2+^ Pro-Bond resin column, and Ma et al., who used a Ni-NTA column and ultrafiltration tube [41].

Once the recombinant azurin had been developed, optimized for production, and successfully purified in a prokaryotic system, the next step was to test its potential cytotoxic effects on three different breast cancer cell lines (MCF-7, MDA-MB-231, and HCC38) and on the normal MCF10A line. The results revealed a cytotoxic effect against all tested breast cancer cell lines, with IC_50_ values of 81, 101, and 122 µg/mL, respectively. The strongest effect was observed for the MCF-7 line; interestingly, no cytotoxic effect was shown for the normal cell line in the tested concentration range.

Our findings are consistent with those of previous research. Aslam et al. showed a significant decrease in MCF-7 cell survival with azurin, with an IC_50_ of 105 μg/mL, with a lessened effect observed in the normal MCF-10F cell line [30]. In contrast, Punj et al. showed that azurin is significantly cytotoxic to the MCF-7 cell line (human breast adenocarcinoma cell line) but, interestingly, less cytotoxic against the p53-negative breast cancer cell line (MDA-MB157) or cell lines with non-functional p53, such as MDD2 and MDA-MB-231 [18]. A similar effect was observed in our present study, where the MDA-MB-231 line was the least sensitive to azurin. Additionally, a cytotoxic effect was demonstrated for the HCC38 line. In another study, Ramachandran et al. showed that azurin induces a significant decrease in the proliferation of T-47D and ZR-75-1 breast cancer cell lines with an IC_50_ of 72 ± 3 µg/mL [19]. A similar effect was observed by Jebur et. al., who confirmed a cytotoxic effect against the MCF-7 line with an IC_50_ of 25.6 µg/mL [45]. These examples show that azurin may be a good candidate for breast cancer therapy and that its negligible toxicity to normal cell lines may protect patients from side effects during the treatment process. In addition, studies have shown it to be effective against other cancer cell lines such as those from colon cancer, melanoma, lung, ovarian, leukemia, bone cancer, and glioblastoma [16,46,47,48,49,50,51].

It has been suggested that azurin has two modes of action on cancer cells, namely via p53 and transmembrane proteins such as the Eph receptor family or P-cadherin. Azurin preferentially enters cancer cells over healthy cells [52], which has been attributed to the p28 domain; this roughly corresponds to the Leu50-Asp77 peptide on azurin, forming an extended amphipathic alpha-helical region connecting the β-sheets and representing the transport domain of the protein. The p28 domain interacts with cholesterol microdomains that are overexpressed on the plasma membrane of cancer cells [53]. This allows azurin to enter cancer cells via the endocytic pathway [54,55]. Once inside the cancer cell, azurin complexes with the p53 protein, preventing its degradation [52]; the resulting complex is imported into the nucleus, where p53 can increase the expression of pro-apoptotic genes [56] and activate the apoptotic mechanism by releasing cytochrome c into the cytosol [57,58]. Our in silico molecular docking simulations further substantiate this mechanism, predicting a stable and energetically favorable complex between azurin and the p53 DNA-binding domain, driven predominantly by strong electrostatic interactions. In this study, we directly substantiated the involvement of the mitochondrial pathway by demonstrating that azurin treatment leads to a significant loss of mitochondrial membrane potential (ΔΨm). This depolarization is a critical upstream event that permeabilizes the mitochondrial membrane, facilitating the release of cytochrome c and initiating the caspase cascade. Our findings, therefore, provide a mechanistic link between azurin’s interaction with cellular components and the subsequent activation of apoptosis observed through increased caspase-3/7 activity, ROS generation, and DNA damage. Our results showed that azurin can induce apoptosis through the mitochondrial pathway by altering the expression of apoptotic genes and proteins, thus increasing the level of ROS, DNA damage, and the level of caspase-3/7 activity. This mechanism has been noted in previous studies that recommend azurin as a good potential candidate for the treatment of breast cancer. Punj et al. showed that azurin increases intracellular levels of Bax and p53 in the nucleus, leading to the release of mitochondrial cytochrome c into the cytosol. This results in the activation of the caspase cascade (including caspase-7 and caspase-9) and the initiation of apoptosis in breast cancer cells. Furthermore, extensive DNA breaks have been noted in azurin-treated cells, supporting the hypothesis that azurin-induced apoptosis involves activation of caspases [18]. In addition, Ghasemi-Dehkordi et al. report that azurin prevents the growth and proliferation of MCF-7 breast cancer cells through control of cell cycle genes and an increase in the proapoptotic genes (BAK, FAS, and BAX) and a decrease in cyclin-D1 (a cell cycle regulator) [9]. In turn, Ramachandran et al. showed that azurin induces apoptosis in T-47D and ZR-75-1 breast cancer cells and mediates growth arrest of these cells through cell cycle arrest in the sub-G1 phase, ROS generation, up-regulation of p53, and up- and down-regulation of pro- and anti-apoptotic proteins, as well as DNA fragmentation; it was also observed that the concentration of azurin required to inhibit cell growth varied depending on the cell line tested. These results are consistent with our findings and confirm that azurin can induce apoptosis by generating reactive oxygen species (ROS), increasing DNA damage and altering apoptotic gene levels, with the effect being dependent on the cell line [19]. Finally, Bernardes et al. showed that azurin attenuates invasion and FAK/Src signaling in MCF-7 breast cancer cell models with P-cadherin overexpression and can therefore probably be used therapeutically to treat poor prognosis breast cancer with P-cadherin overexpression [59].

These findings are in line with ours and confirm the antitumor character of azurin in different breast cancer cell lines. However, it is worth noting that its effect varies from line to line, which may reflect the observed differences in sensitivity. While these in vitro findings are promising and demonstrate the selective pro-apoptotic potential of recombinant azurin against the tested breast cancer cell lines, we acknowledge that this study, as an initial investigation, has inherent limitations. The current work focused primarily on establishing a robust production system for recombinant azurin and characterizing its fundamental anticancer effects and mechanisms of action at the cellular level. To translate these encouraging in vitro results into tangible therapeutic strategies, further comprehensive research is indispensable. Future studies should rigorously address the challenge of efficient and tumor-selective delivery of azurin to cancer cells in vivo. This could involve exploring various nanocarrier systems, peptide-based delivery vectors, or other advanced formulation strategies designed to enhance intracellular uptake, improve pharmacokinetic profiles, and minimize potential off-target effects. Investigating alternative approaches, such as the feasibility of expressing azurin directly within cancer cells using, for instance, mRNA-based technologies or viral vectors, also presents a valuable avenue for future exploration. Ultimately, the therapeutic efficacy and safety of recombinant azurin must be rigorously validated in well-designed preclinical in vivo animal models of breast cancer. Such studies would be essential to assess its antitumor activity in a more complex biological environment, determine its therapeutic index, and understand its pharmacokinetics and biodistribution before any consideration for clinical translation. The foundational data presented in this manuscript provide a strong rationale and a crucial stepping stone for pursuing these important and necessary subsequent stages of research.

## 4. Materials and Methods

### 4.1. In Silico Protein–Protein Docking

To investigate the structural basis of the interaction between azurin and the p53 tumor suppressor protein, information-driven docking simulations were performed using the HADDOCK 2.4 (High Ambiguity Driven DOCKing) web server [60,61].

The atomic coordinates for the docking partners were prepared as follows. The structure of *Pseudomonas aeruginosa* azurin was based on the PDB entry 4AZU (chain A), and the structure of the human p53 DNA-binding domain (DBD) was based on PDB entry 1TUP (chain A). To ensure structural integrity and standardized nomenclature, both structures were first submitted to the PyMOL software (The PyMOL Molecular Graphics System, Version 3.1, Schrödinger, LLC, New York, NY, USA) to generate clean PDB files containing only the protein chain and its essential cofactors. Specifically, all non-protein molecules, including solvent, DNA, and additional protein chains, were removed. The charges of the copper ion (Cu) in azurin and the zinc ion (Zn) in the p53 DBD were explicitly defined as +2 in the final PDB files.

The docking simulations were guided by defining Ambiguous Interaction Restraints (AIRs) based on previously published data. For azurin, residues constituting the p28 domain (Leu50–Asp77) were defined as “active” residues. For the p53 DBD, key surface residues implicated in protein–protein interactions (Lys120, Cys175, Ser241, Arg248, Met249, Arg273, Cys277, Arg280) were designated as “active”. “Passive” residues, defined as all surface amino acids surrounding the active sites, were automatically selected by the server.

Docking was performed using the standard HADDOCK protocol with default parameters. This protocol consists of three main stages: (1) rigid-body energy minimization (it0), (2) semi-flexible simulated annealing in torsion angle space (it1), and (3) a final refinement in explicit water solvent (itw).

The resulting structures were clustered using a fractional common contact (FCC) similarity matrix with a 0.6 cutoff. The generated clusters were ranked according to their mean HADDOCK score, a weighted sum of van der Waals, electrostatic, desolvation, and restraint violation energies. The top-ranked cluster, characterized by the most favorable HADDOCK score and a significant Z-score, was selected for further analysis. Visualization and structural analysis of the representative docked complex were performed using The PyMOL Molecular Graphics System, Version 2.5 (Schrödinger, LLC).

### 4.2. Recombinant Vector Construction

The recombinant bacterial expression vector was prepared according to the following procedure. Plasmid pT7-MAT-Tag-2 (Sigma, St. Louis, MO, USA) was subjected to simultaneous hydrolysis with HindIII and KpnI (EURx, Gdansk, Poland) restriction enzymes to prepare it for the azurin encoding sequence. The sequence encoding azurin (Uniprot ID: B3EWN9) was excised from plasmid pEX-K168-azu (Eurofins Genomics, Ebersberg, Germany) to prepare it for cloning into the expression vector. Digestion with restriction enzymes was performed as follows: 1 µg of plasmid DNA, 5 µL of 10× Buffer ONE, 0.5 µL BSA [100×], 1 U of each restriction enzyme, and sterile H_2_O was added to a final volume of 50 µL. Hydrolysis was carried out at 37 °C for 1 h. The hydrolysis products were then separated on a 1.5% LMP agarose gel (EURx, Gdansk, Poland). DNA fragments were stained with ethidium bromide and then isolated from the gel using the Agarose-Out DNA Purification Kit (EURx, Gdansk, Poland). The concentration of DNA fragments for ligation was assessed spectrophotometrically using a Qubit 4 fluorometer with a dsDNA BR Assay Kit (Invitrogen, Life Technologies, Waltham, MA, USA). The ligation reaction was performed using the Liga5 ligation kit (A&A Biotechnology, Gdansk, Poland). The reaction mixture was used to transform competent *E. coli* DH5αa cells using an *E. coli* Transformer Express kit (A&A Biotechnology, Gdansk, Poland) according to the manufacturer’s instructions. A 5 mL portion of LB medium was inoculated with bacteria from a single colony and then incubated overnight at 37 °C with vigorous shaking. Subsequently, 1 mL of the overnight culture was inoculated with 100 mL of medium, which was incubated in a shaker with vigorous shaking for 3 h at 37 °C. Bacterial growth was monitored by periodically measuring the optical density at 600 nm (OD600) until a value of 0.5 was reached. The culture was cooled for 15 min and then centrifuged for 5 min at 3500 RPM at 4 °C. The supernatant was removed, and the pellet was gently resuspended in 2 mL of S1 Express solution. Then, 2 mL of S2 Express solution was added and gently mixed. The bacterial suspension prepared in this way was cooled on ice for 15 min and aliquoted in 100 µL portions into sterile Eppendorf type tubes. Transformation was carried out as follows: 1 µL of ligation mixture was added to competent bacterial cells and gently mixed. Cells were incubated on ice for 5 min and then transferred into a mixture of 1 mL of SOC medium heated to 37 °C. The culture was incubated for 45 min with vigorous shaking at 37 °C. The bacteria were then vortexed, most of the supernatant removed, and the cells were seeded onto LB selection medium plates. The plates were incubated overnight at 37 °C. Transformed *E. coli* bacterial cells were cultured on agar-solidified (1.5%) LB medium (Tryptone 10 g/L, NaCl 10 g/L, Yeast extract 5 g/L, pH 7.5) supplemented with 100 mg/L ampicillin (Duchefa Biochemie, Haarlem, The Netherlands, Cat. Number A0104.0005). The plasmid DNA isolated from these cells was then checked for construct validity using restriction hydrolysis and PCR.

### 4.3. Confirmation of Vector Construction

The correctness of the construction of the recombinant vector containing the sequence encoding azurin was checked by re-hydrolyzing it with the restriction enzymes previously used for cloning. Following this, 200 ng of plasmid DNA was digested with HindIII and KpnI endonucleases (EURx, Gdansk, Poland). The sequence encoding azurin in the recombinant plasmid was confirmed by PCR using a PCR Mix Plus Red kit (A&A Biotechnology, Gdansk, Poland) in a Biometra UNO II thermocycler (Biometra, Göttingen, Germany), according to the manufacturer’s instructions. The reaction was carried out in 25 µL with 12.5 µL of 2xPCRMixPlusRed, 0.2 µM of each primer, and 1 µL of template DNA. The following primer pair was used for the PCR reaction (5′-GCCGAATGTTCCGTTG-3′ and 5′-ACTTTAACGTCAACGTTCCTTT-3′). The PCR conditions were as follows: 5 min at 94 °C, followed by 30 cycles of 1 min at 94 °C, 1 min at 48 °C, and 1 min at 72 °C. Finally, the mixture was left for 5 min at 72 °C. The PCR products were identified by 1.5% agarose gel electrophoresis.

### 4.4. E. coli Cells Transformation and Production of Recombinant Azurin

The recombinant vector containing the azurin gene was introduced into chemically competent *E. coli* BL21(DE3) Rosetta T1^R^ (Novagen Inc., Madison, WI, USA) cells prepared with an *E. coli* Transformer Express kit (A&A Biotechnology, Gdansk, Poland) according to the manufacturer’s instructions. In brief, bacteria derived from a single colony on a Petri dish were used to initiate an overnight culture in LB medium; following this, 2 mL of the bacterial suspension was used to inoculate a volume of 100 mL. The bacteria were cultured at 37 °C with vigorous shaking until OD_600_ = 0.5. The culture was then cooled for 15 min on ice, centrifuged (5300 rpm, 4 °C), and the bacterial pellet was resuspended in 2 mL of S1E solution. Then, 2 mL of S2E solution was added, the suspension was cooled on ice for 15 min, and then aliquoted in 100 µL portions into Eppendorf tubes. The prepared cells were subjected to genetic transformation with the recombinant plasmid. Briefly, 50 ng of plasmid DNA was added to the cells, mixed, and then left on ice for 30 min. The bacterial cells were then transferred to a 42 °C water bath for 60 s. After this time, the cells were incubated in ice for 2 min. Then, the entire bacterial suspension after transformation was transferred to 1 mL of LB liquid medium and shaken at 37 °C for one hour. After this time, the bacteria were seeded on LB medium with ampicillin (Duchefa Biochemie, Cat. Number A0104.0005) and chloramphenicol (Duchefa Biochemie, Cat. Number C0113.0025) (34 and 100 mg/L, respectively).

The production of recombinant azurin in bacterial cells was carried out as follows. Bacteria from single colonies selected on LB medium supplemented with chloramphenicol and ampicillin were used to establish the initial culture. The culture was conducted at 37 °C with vigorous shaking for 16 h. Subsequently, 2 mL of culture was transferred to 100 mL of TB medium (yeast extract 24 g/L, tryptone 20 g/L, glycerol 4 mL/L, phosphate buffer: 0.017 M KH_2_PO_4_, 0.072 M K_2_HPO_4_). The medium without phosphate buffer was prepared in 900 mL of deionized water and autoclaved for 20 min at 15 psi. After cooling the medium to 50 °C, sterile phosphate buffer was added to achieve the appropriate final concentration. Bacteria were cultured to OD_600_ = 0.5, and IPTG (concentration 0.1–1.0 mM) was added to optimize recombinant protein production. After induction, bacteria were cultured at 25 °C or 37 °C for further optimization for 4–12 h with vigorous shaking (200–225 rpm). The bacteria were then centrifuged to remove the culture medium, and the protein isolation procedure was performed.

### 4.5. E. coli Protein Isolation

Bacteria from the culture were harvested by centrifugation at 5000× *g* for 10 min. The culture medium was then removed, and CelLytic B (Sigma-Aldrich, St. Louis, MO, USA) was added at 10 mL per gram of cell pellet with lysozyme (0.2 mg/mL) and Protease Inhibitor Cocktail (Merck-Sigma). The sample was mixed thoroughly until the entire pellet was completely suspended. The suspension was incubated with shaking at room temperature for 15 min and then centrifuged at 16,000× *g* for 10 min to remove insoluble material. The supernatant, which contained the isolated proteins, was transferred to a new tube.

### 4.6. Recombinant Azurin Purification

Recombinant azurin was purified using HIS-Select^®^ Nickel Affinity Gel (Merck-Sigma). First, the column was washed with two volumes of deionized water followed by three volumes of equilibration buffer (50 mM sodium phosphate, pH 8.0, with 0.3 M sodium chloride and 10 mM imidazole). Next, the clarified crude extract was applied to the column, and then the column was washed with wash buffer until a stable A_280_ value equal to that of the wash buffer was obtained. The recombinant purified azurin was eluted from the column using five volumes of elution buffer (50 mM sodium phosphate (pH 8.0), with 0.3 M NaCl and 250 mM imidazole). The recombinant azurin was concentrated and washed using a Vivaspin 6 centrifugal concentrator (Vivaspin 6 with an MWCO of 5 kDa, Sartosius, Göttingen, Germany). The final protein was filtered using Whatman Puradisc 30/0.2 CA-S syringe filters (Sigma-Aldrich, Dorset, UK).

### 4.7. SDS-PAGE and Western Blot Analysis

SDS-PAGE analysis was performed using a 15% separating gel on a Mini-PROTEAN 3 system (Bio-Rad, Hercules, CA, USA). Briefly, the total lysates of *E. coli* cells transformed and untransformed with the recombinant plasmid and the purified protein were loaded onto the gel. The proteins were separated by electrophoresis at 150 V for one hour and stained with Coomassie Brilliant Blue G-250.

The recombinant azurin was identified by Western blotting. After electrophoresis, the proteins were transferred to a polyvinylidene difluoride (PVDF) membrane by wet transfer in the Mini Trans-Blot^®^ Electrophoretic Transfer Cell system (25 mM Tris, pH 8.3, 192 mM glycine, with 20% MeOH and 0.1% SDS). The PVDF membrane was first wetted in 100% methanol for 10 s and then transferred to the transfer buffer for three minutes before further processing. The properly assembled setup was placed in the apparatus, and electrotransfer onto the membrane was performed overnight in the transfer buffer at 4 °C, at a constant current of 20 mA. After transfer, the membrane was washed three times in TBST buffer (20 mM Tris, pH 7.5, 150 mM NaCl, 0.1% Tween 20) for 10 min; then, the membrane was incubated in blocking buffer (3% bovine serum albumin in TBS) for 1.5 h at room temperature. Then, anti-His antibody (HRP, Genetex, Irvine, CA, USA, Cat. number: GTX628914-01) was added (1:10,000 dilution) in blocking buffer and incubated for two hours. The membrane was then washed three times in TBST buffer for 5 min and then transferred to DAB solution (50 mg of 3,3′-diaminobenzidine in 100 mL of TBS supplemented with 10 µL of 30% H_2_O_2_) and incubated until the desired development was achieved.

### 4.8. Cell Culture Models

The MCF7 (human breast cells, mammary gland, adenocarcinoma; HTB-22™), MDA-MB-231 (human breast cells, mammary gland, adenocarcinoma; HTB-26), and HCC38 (human breast cells, duct; mammary gland, carcinoma, ductal; CRL-2314) and the normal MCF10A cell lines (breast; mammary gland; CRL-10317) were obtained from the American Type Culture Collection (ATCC, Manassas, VA, USA). The cell lines listed above were cultured in a humidified 5% CO_2_ atmosphere at 37 °C (New Brunswick Galaxy^®^170R CO_2_ Incubator, Hamburg, Germany). Monolayer cells were grown in Eagle’s Minimum Essential Medium (EMEM; Biowest—MCF-7), Dulbecco’s Modified Eagle Medium (DMEM, Biowest—MDA-MB-231), and RPMI-1640 medium (HCC38) supplemented with 10% fetal bovine serum (Biowest), as recommended by the manufacturer. These cell culture media were also supplemented with penicillin (100 U/mL) and streptomycin (100 μg/mL). MCF10A cells were cultured in MEGM™ Mammary Epithelial Cell Growth Basal Medium (Lonza, Basel, Switzerland, CC-3151) with MEGM SingleQuots^TM^ Supplements (Lonza, CC-4136) containing bovine pituitary extract (BPE), human epidermal growth factor (hEGF), insulin, gentamicin sulfate/amphotericin (GA-1000), and hydrocortisone.

### 4.9. MTT

The MTT (3-(4,5-dimethylthiazol-2-yl)-2,5 tetrazolium bromide) assay was used to measure the cytotoxic effect after azurin treatment. The MCF-7, MDA-MB-231, HCC38, and MCF10A cells at 80–85% confluence were detached using TrypLE™ Express Enzyme without phenol red (Gibco, Grand Island, NY, USA) and then were seeded in 96-well plates at a density of 10^4^ cells/well (three replicates) and allowed to adhere and grow in a CO_2_ (5%) incubator at 37 °C overnight. The following day, the medium was removed, and the cells were treated with azurin (at concentrations from 0 μg/mL to 100 μg/mL) for 24 h. The medium was then removed, and the cells were incubated with 100 µL MTT solution (5 mg/mL MTT in PBS) for four hours. MTT formazan was measured spectrophotometrically at 590 nm in a microplate reader (Synergy HT, BIO-TEK, Winooski, VT, USA). The IC_50_ value indicates the concentration at which 50% of the cells remain alive compared to the control sample and is hence used as an indicator of cell viability.

### 4.10. Gene Expression

MCF-7, MDA-MB-231, and HCC38 cancer cells were incubated with azurin at the IC_50_ concentration for 24 h. Gene expression was analyzed using TaqMan probe-based real-time PCR. The TaqMan probes used in this study were as follows: Hs00608023_m1 (*BCL2*), Hs00180269_m1 (*BAX*), Hs00153349_m1 (*TP53*), Hs00234387_m1 (*Casp3*), Hs01018151_m1 (*Casp8*), Hs00962278_m1 (*Casp9*), and Hs00559441_m1 (*Apaf1*). The 18S ribosomal RNA (rRNA) gene (Assay ID: Hs99999901_s1) was used as the endogenous reference gene for normalization of gene expression data (Life Technologies, Carlsbad, CA, USA). RT-PCR was performed using TaqMan™ Gene Expression Master Mix (Thermo Fisher Scientific, Waltham, MA, USA). The CFX96™ Real-Time PCR Detection System (Bio-Rad Laboratories, Hercules, CA, USA) was used. The RT-PCR protocol was as follows: 10 min of polymerase activation at 95 °C, followed by 40 cycles of denaturation at 95 °C for 30 s and annealing/extension at 60 °C for 60 s. Samples were run in triplicate. The ΔΔCt method [62] was used to calculate relative gene expression.

### 4.11. ELISA Test

All tested cancer cells were incubated with azurin at the IC_50_ concentration for 24 h. The concentrations of BAX, BCL-2, p53, and APAF-1 were determined by ELISA using commercial ELISA kits (Cloud-Clone Corp., Houston, TX, USA), according to the manufacturer’s protocol. Briefly, cell lysates and all reagents (standards and blank) were prepared according to the manufacturer’s guidelines by adding 100 μL of each standard dilution, blank, and sample to the appropriate wells. Incubation was performed for 1 h at 37 °C, after which the liquid was removed from each well, and 100 μL of Detection Reagent A working solution was added to each well. The covered plates were incubated for 1 h at 37 °C. The solution was removed, the wells were washed with the appropriate volume of 1× wash solution added to each well, and then the plates were left to stand for 2 min. The remaining liquid was then removed from all wells, and the wells were washed three times. After the final wash, any remaining wash buffer was thoroughly removed. Then, 100 μL of Detection Reagent B working solution was added to each well and incubated for 30 min at 37 °C; the wells were washed five times, and 90 μL of substrate solution was added to each well. The sealed plates were incubated for 15 min at 37 °C in the dark, after which 50 μL of stop solution was added to each well. Measurement was performed using a microplate reader (Synergy HT, BIO-TEK, Winooski, VT, USA) at 450 nm.

### 4.12. Genotoxicity Assay (Comet Assay)

The genotoxicity of azurin was determined by the comet assay. The final concentration was adjusted to 1 × 10^5^ cells/mL of MCF-7, MDA-MB-231, and HCC38 cancer cells in each sample. The previously determined IC_50_ concentrations of azurin were used in the test. The comet assay was performed under alkaline conditions (pH > 13) as described previously [17]. The cells were incubated with azurin at 37 °C for one hour. The samples were then centrifuged (182× *g*, 15 min, 4 °C). They were then resuspended in 0.75% L-MMP agarose, layered on slides precoated with 0.5% N-MMP agarose, and lysed for one hour at 4 °C in a buffer consisting of 2.5 M NaCl, 1% Triton X-100, 100 mM EDTA, and 10 mM Tris, pH 10. After lysis, electrophoresis was performed in an electrophoresis solution containing 300 mM NaOH and 1 mM EDTA to allow the DNA to unwind. Electrophoresis was performed for 20 min under an electric field strength of 0.73 V/cm (300 mA). The slides were neutralized, air dried overnight, and stained with 1 µg/mL DAPI.

The comets were observed at 200× magnification using a fluorescence microscope (Nikon Eclipse Ci-H600L, Tokyo, Japan) connected to a video camera and a PC-based image analysis system (Lucia-Comet v. 7.0, Laboratory Imaging, Prague, Czech Republic). For analysis, 50 images were randomly selected per sample. The percentage of DNA in the comet tails was used to measure DNA damage. Aliquots of the same sample were used for two parallel assays. The mean DNA damage was calculated from a total of 100 cells.

### 4.13. Intracellular ROS Measurement

Dichlorofluorescein diacetate (H_2_DCFDA, Invitrogen™, Waltham, MA, USA), a membrane-permeant fluorescent probe widely used for monitoring intracellular ROS production, was used for the analysis of intracellular ROS generation. The MCF-7, MDA-MB-231, and HCC38 cancer cells used for the analysis were seeded in 96-well plates at 1 × 10^5^ cells/well in 50 μL culture medium. The cells were cultured at 37 °C for 12 h in an environment containing 5% CO_2_. The cells were then treated with azurin at IC_50_ concentrations and incubated 2 to 24 h. Untreated cells were used as controls. After the treatment, the cells were incubated with 5 µM DCFH-DA (prepared in HBSS buffer) at 37 °C for 45 min. Fluorescence was measured at an excitation wavelength of 480 nm and an emission wavelength of 510 nm using a Bio-Tek Synergy HT Microplate Reader (Bio-Tek Instruments, Winooski, VT, USA). The intensity was expressed as DCF fluorescence.

### 4.14. Caspase-3/7 Activity

Caspase-3/7 activation was evaluated to determine whether azurin promotes cell death in the MCF-7, MDA-MB-231, and HCC38 cell lines. After incubation of the cell lines with azurin, caspase-3/7 activity was determined using a fluorescence microplate reader and CellEvent™ Caspase-3/7 Green Detection Reagent (Invitrogen). Briefly, MCF-7, MDA-MB-231, and HCC38 cells were seeded at 1 × 10^5^ cells/well in 50 µL culture medium in a transparent bottomed 96-well microplate and incubated at 37 °C, 5% CO_2_ to allow cell attachment. Following overnight incubation, the cells were rinsed with 1× PBS to eliminate non-adherent cells, treated with azurin, and incubated for another 24 h under the same conditions. CellEvent Caspase-3/7 Green Detection Reagent was then added at a final concentration of 5 µM. Caspase activity was determined after incubation for 30 min. The fluorescence intensity of the cells was measured at an excitation wavelength of 502 nm and an emission wavelength of 530 nm using a Bio-Tek Synergy HT Microplate Reader. The assay was performed in triplicate for each sample.

### 4.15. Assessment of Mitochondrial Membrane Potential (ΔΨm)

To determine whether azurin-induced apoptosis involves the mitochondrial pathway, changes in the mitochondrial membrane potential (ΔΨm) were evaluated using the ratiometric fluorescent probe JC-1 (5,5′,6,6′-tetrachloro-1,1′,3,3′-tetraethylbenzimidazolylcarbocyanine iodide). This lipophilic cationic dye accumulates in the mitochondria in a potential-dependent manner. In healthy cells with normally polarized mitochondria and a high ΔΨm, JC-1 forms aggregates that emit intense red fluorescence. However, following the loss of membrane potential, a key event in the early stages of apoptosis, the dye dissociates into monomers, and its fluorescence emission shifts from red to green.

For the assay, MCF-7, MDA-MB-231, and HCC38 cells were seeded in black 96-well plates with transparent bottoms at a density of 1 × 10^5^ cells/well. After overnight incubation to ensure adherence, the cells were treated with recombinant azurin at the respective IC_50_ concentration for 24 h. Following treatment, the cells were incubated with 5 μM JC-1 in HBSS for 30 minutes at 37 °C in a CO_2_ incubator, protected from light. Subsequently, the cells were washed twice with HBSS to remove any background fluorescence.

The fluorescence was quantified using a Bio-Tek Synergy HT Microplate Reader. The intensity of red fluorescence (J-aggregates) was measured at an excitation/emission of 530 nm/590 nm, and the intensity of green fluorescence (JC-1 monomers) was measured at an excitation/emission of 485 nm/538 nm. The results from three independent experiments were expressed as the ratio of red-to-green fluorescence intensity, and the values were normalized against the untreated control cells. A decrease in this ratio was considered indicative of mitochondrial membrane depolarization.

### 4.16. General Toxicity Assessment—Brine Shrimp Lethality Assay (BSLA)

The general toxicity of recombinant azurin was evaluated using the brine shrimp (*Artemia salina*) lethality assay. *Artemia salina* eggs were hatched in artificial seawater (3.8% w/v sea salt in deionized water) under constant illumination for 48 h at room temperature to obtain motile nauplii. The bioassay was conducted in a 24-well microplate, to which approximately 10–15 nauplii were transferred into each well containing artificial seawater. Recombinant azurin, previously dissolved in its storage buffer, was added to the wells to achieve a range of final concentrations (e.g., 10, 50, 100, 200, and 500 µg/mL). A negative control containing only nauplii in seawater, a vehicle control containing the protein buffer at a volume corresponding to the highest concentration tested, and a positive control using potassium dichromate (K_2_Cr_2_O_7_) at a final concentration of 1 mg/mL were included to validate the assay. The plates were then incubated for 24 h at approximately 25 °C. After the incubation period, the number of dead and surviving nauplii in each well was counted, and the percentage of mortality was calculated for each concentration. The entire experiment was performed in triplicate, with each test condition and control group replicated at least three times.

### 4.17. Data Analysis

All quantitative data are presented as the mean ± standard deviation (SD) from at least three independent experiments. Statistical analyses were performed using GraphPad Prism 9 software (GraphPad Software, La Jolla, CA, USA). For comparisons involving two groups (e.g., a single treatment group versus a control), the data were analyzed using the Student’s *t*-test, or the Mann–Whitney U test if the data did not follow a normal distribution. For dose–response experiments or comparisons involving multiple treatment groups against a single control (such as the MTT and brine shrimp lethality assays), a one-way analysis of variance (ANOVA) was employed, followed by Dunnett’s post hoc test. A *p*-value of less than 0.05 was considered statistically significant. The levels of significance are indicated in the figures as * *p* < 0.05, ** *p* < 0.01, and *** *p* < 0.001.

## 5. Conclusions

The study describes the design and evaluation of a constructed recombinant pT7-MAT-Tag-2-Azu vector for the production of recombinant azurin in *E. coli* cells. The results showed that azurin exhibited cytotoxic effects on the tumor cells tested, while showing minimal toxicity to normal cells and in an in vivo assay on brine shrimp. Additionally, the use of recombinant azurin led to increased levels of ROS and significant DNA damage in the tumor cells with a decrease in mitochondrial membrane potential. Noteworthy is the selective cytotoxicity of azurin: it appears to be associated with preferential entry into tumor cells, induction of apoptosis via mitochondrial pathways, which our in silico docking simulations support by predicting a stable and energetically favorable complex with the p53 protein, and increased expression of proapoptotic genes while decreasing expression of anti-apoptotic genes and corresponding protein levels. Biotechnological production of recombinant azurin may offer exciting opportunities to create new effective anticancer drugs or components to complement traditional therapies. The ability to manipulate genes and create fusion proteins could become an effective solution for diseases that are currently difficult to overcome. Our observations suggest that azurin could be a promising candidate for future therapeutic use, particularly in targeting cancer cells with minimal side effects on normal cells.

## Data Availability

The datasets presented in the current study are available from the corresponding author upon reasonable request.
